# Pancreatic Adenocarcinoma Presenting as a Pathologic Femoral Fracture: A Rare Initial Manifestation

**DOI:** 10.7759/cureus.100772

**Published:** 2026-01-04

**Authors:** Genan Arman, Matt Demir, Robert Mocharnuk

**Affiliations:** 1 Internal Medicine, Southern Illinois University School of Medicine, Springfield, USA; 2 Hematology/Oncology, Southern Illinois University School of Medicine, Springfield, USA

**Keywords:** endoscopic ultrasound (eus), eus fna, lytic lesion, pancreas lesion, pancreatic metastasis, pancreatic tumors, pathologic hip fracture

## Abstract

Pancreatic tail cancers often manifest with vague, nonspecific symptoms, contributing to delayed diagnosis. Bone metastasis from pancreatic adenocarcinoma is uncommon, and presentation with a pathologic fracture is especially atypical. We present the case of a 62-year-old man with obesity, long-standing tobacco use, and regular alcohol consumption who developed an atraumatic femoral fracture. Imaging revealed a destructive lytic lesion in the femur and a pancreatic tail mass with pulmonary nodules. Endoscopic ultrasound-guided fine-needle aspiration biopsy established the diagnosis of pancreatic adenocarcinoma. The patient had an open reduction fixation by orthopedic surgery followed by oncology referral for systemic therapy. This case underscores the importance of considering occult malignancy in atypical fractures and highlights that gastrointestinal malignancies, though infrequent, can metastasize to bone. Early recognition may facilitate prompt diagnosis and improve multidisciplinary management.

## Introduction

Tumors in the pancreatic head are often detected earlier due to symptoms such as obstructive jaundice, whereas lesions in the tail typically go unnoticed until metastatic spread is evident [1,2]. Common metastatic sites include the liver, lungs, and peritoneum; however, skeletal metastases are rare and underreported, with an incidence ranging from 5% to 20% in autopsy series [2,3]. Presentation with bone pain or a pathologic fracture as the initial sign of pancreatic cancer is exceedingly uncommon [4]. This case illustrates such an atypical presentation, emphasizing the need for heightened clinical suspicion and a systematic diagnostic approach when evaluating unexplained fractures.

## Case presentation

A 62-year-old man with hypertension, hyperlipidemia, obesity, chronic chewing tobacco use (2-3 tins/week for 40 years, which is equivalent roughly to 46-69 pack-years of smoking), and alcohol intake (one-fifth of liquor weekly, which is equivalent to 17 standard drinks per week) presented with sudden worsening of chronic right thigh pain after standing from a seated position. He was unable to bear weight due to severe pain. He denied any yellowing of his skin or eyes, dark urine, light stools, persistent abdominal or back pain, unexplained weight loss, loss of appetite, fatigue, nausea, or vomiting. His vitals were stable on arrival, and his physical exam was unremarkable (no jaundice, muscle wasting, abdominal tenderness, palpable masses in the abdomen, ascites, or palpable lymph nodes). 

**Figure 1:**
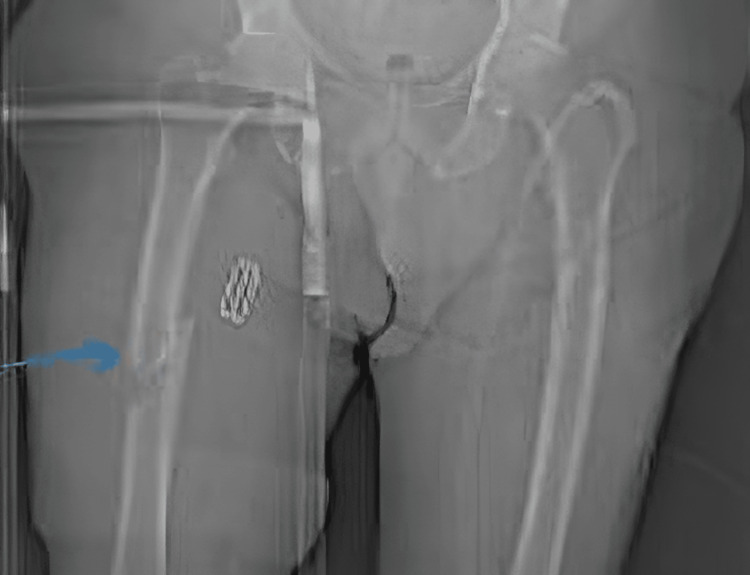
CT of the right thigh without contrast showing a pathologic fracture through a lytic lesion of the mid-femoral shaft Arrows indicate the cortical destruction and fracture line. CT: computed tomography

X-ray of the right lower extremity demonstrated a mid-femoral fracture through a lytic lesion, and computed tomography (CT) confirmed a pathologic fracture of the mid-femoral diaphysis (Figure 1).

**Figure 2:**
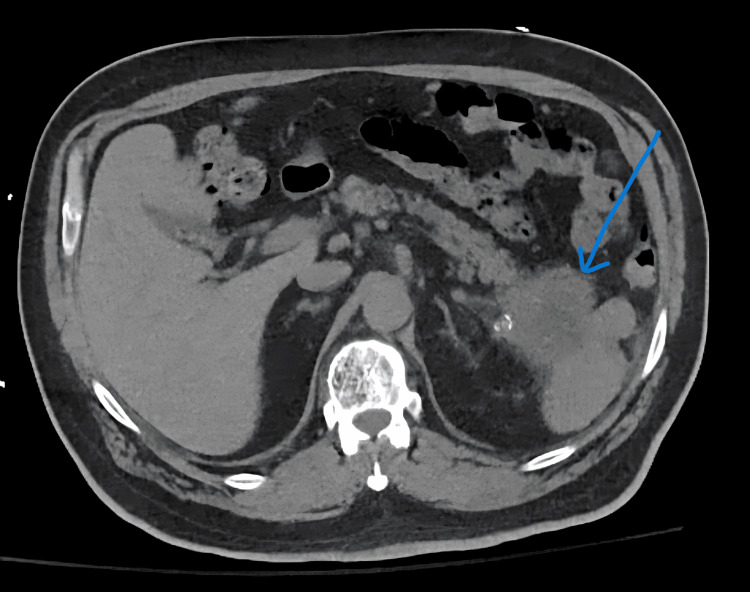
CT of the chest, abdomen, and pelvis showing a 5.7×5.5 cm mass arising from the pancreatic tail and invading the spleen CT: computed tomography

**Figure 3:**
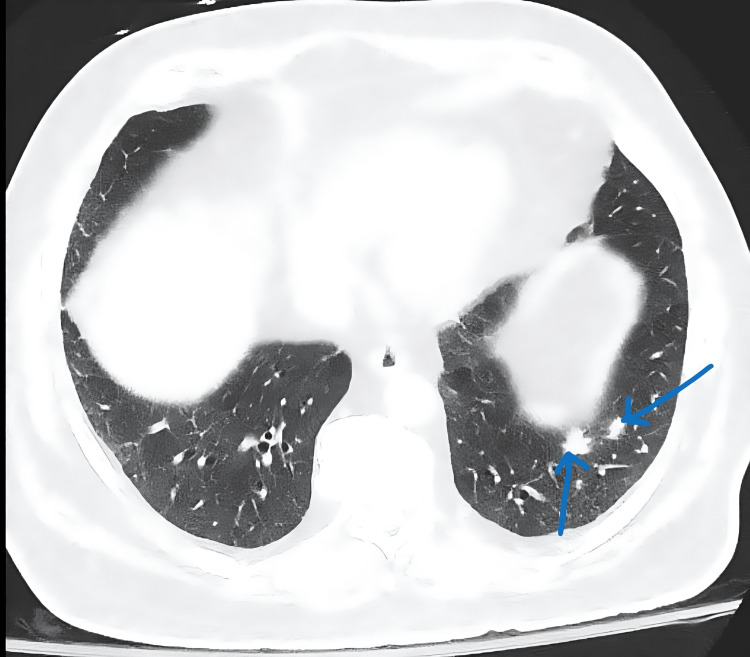
CT of the chest showing multiple pulmonary nodules suspicious for metastasis Arrows highlight representative metastatic nodules in the lung fields. CT: computed tomography

Orthopedic surgery performed open reduction and internal fixation. Given concern for malignancy, further imaging was obtained. CT of the chest, abdomen, and pelvis revealed a 5.7×5.5 cm mass arising from the pancreatic tail (Figure 2) and multiple pulmonary nodules suspicious for metastasis (Figure 3).

**Figure 4:**
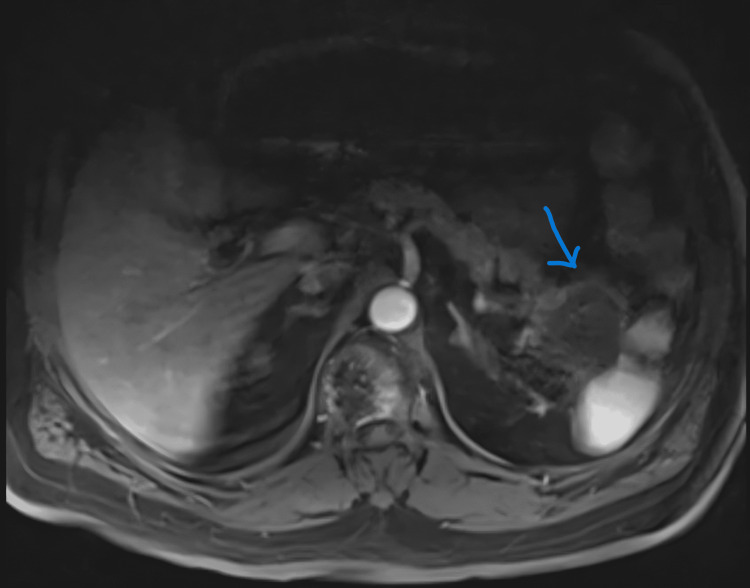
MRI of the abdomen demonstrating a 5.4 cm pancreatic tail mass with splenic invasion Arrows point to the pancreatic lesion and its interface with the spleen. MRI: magnetic resonance imaging

Magnetic resonance imaging (MRI) of the abdomen demonstrated a 5.4 cm pancreatic tail mass with splenic invasion and para-aortic lymphadenopathy (Figure 4).

**Figure 5:**
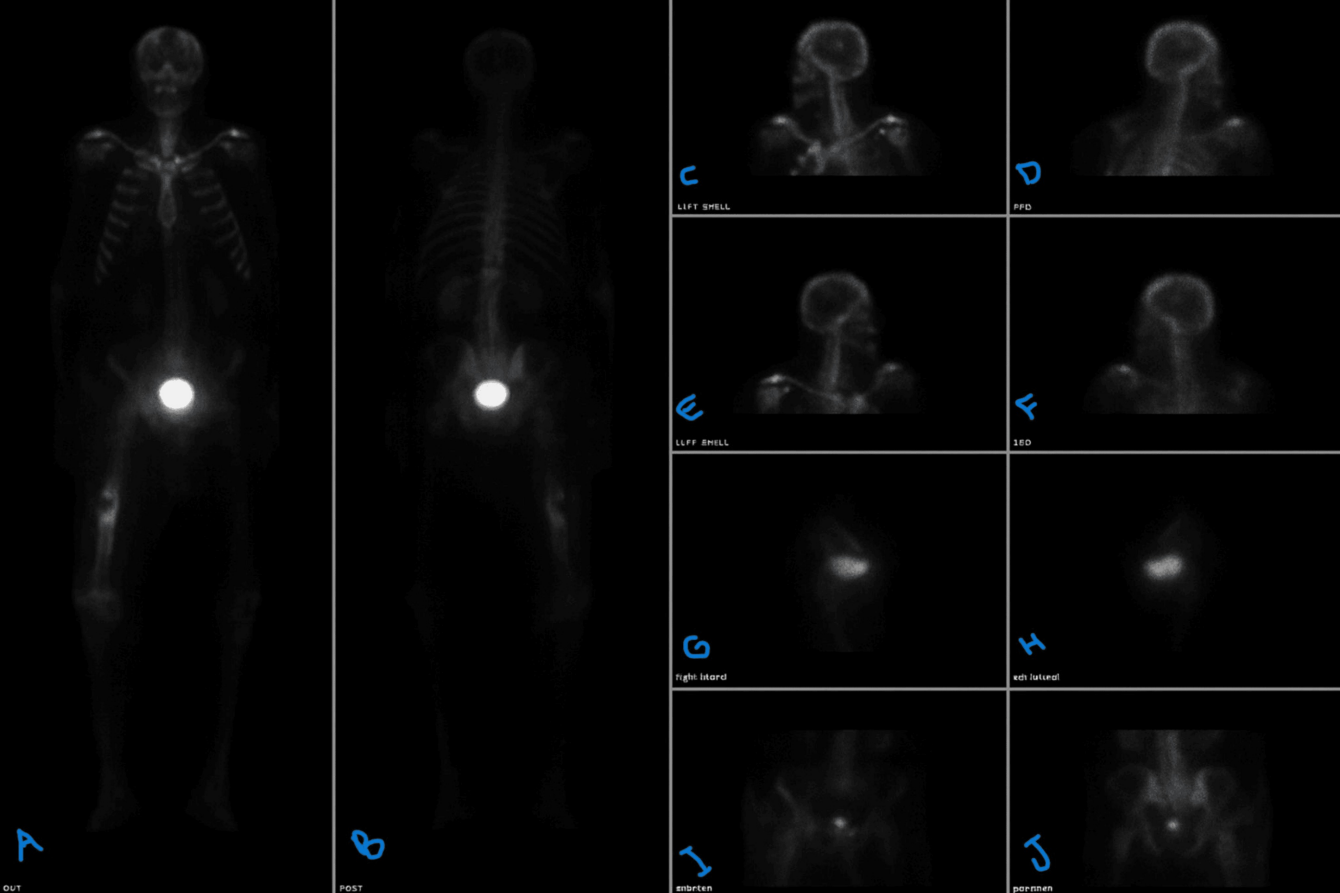
Nuclear bone scan showing a photopenic lesion in the right femur corresponding to the lytic metastasis and increased uptake surrounding the fracture site (A) Anterior whole-body view showing the overall skeletal tracer distribution. (B) Posterior whole-body view highlighting the photopenic region in the right femur. (C) Focused left lateral skull view. (D) Right posterior oblique skull view. (E) Left posterior oblique skull view. (F) Right lateral skull view. (G) Right femur lateral view demonstrating a photopenic lytic lesion with surrounding increased uptake. (H) Posterior pelvis and femur view showing the lesion with reactive uptake at the fracture site. (I) Magnified right femur image illustrating photopenia at the metastatic site. (J) Magnified right femur image highlighting the peripheral increased tracer uptake consistent with a healing or reactive response around the fracture site.

Nuclear bone scan showed a photopenic lesion in the right femur corresponding to the lytic metastasis and increased uptake surrounding the fracture site (Figure 5).

**Figure 6:**
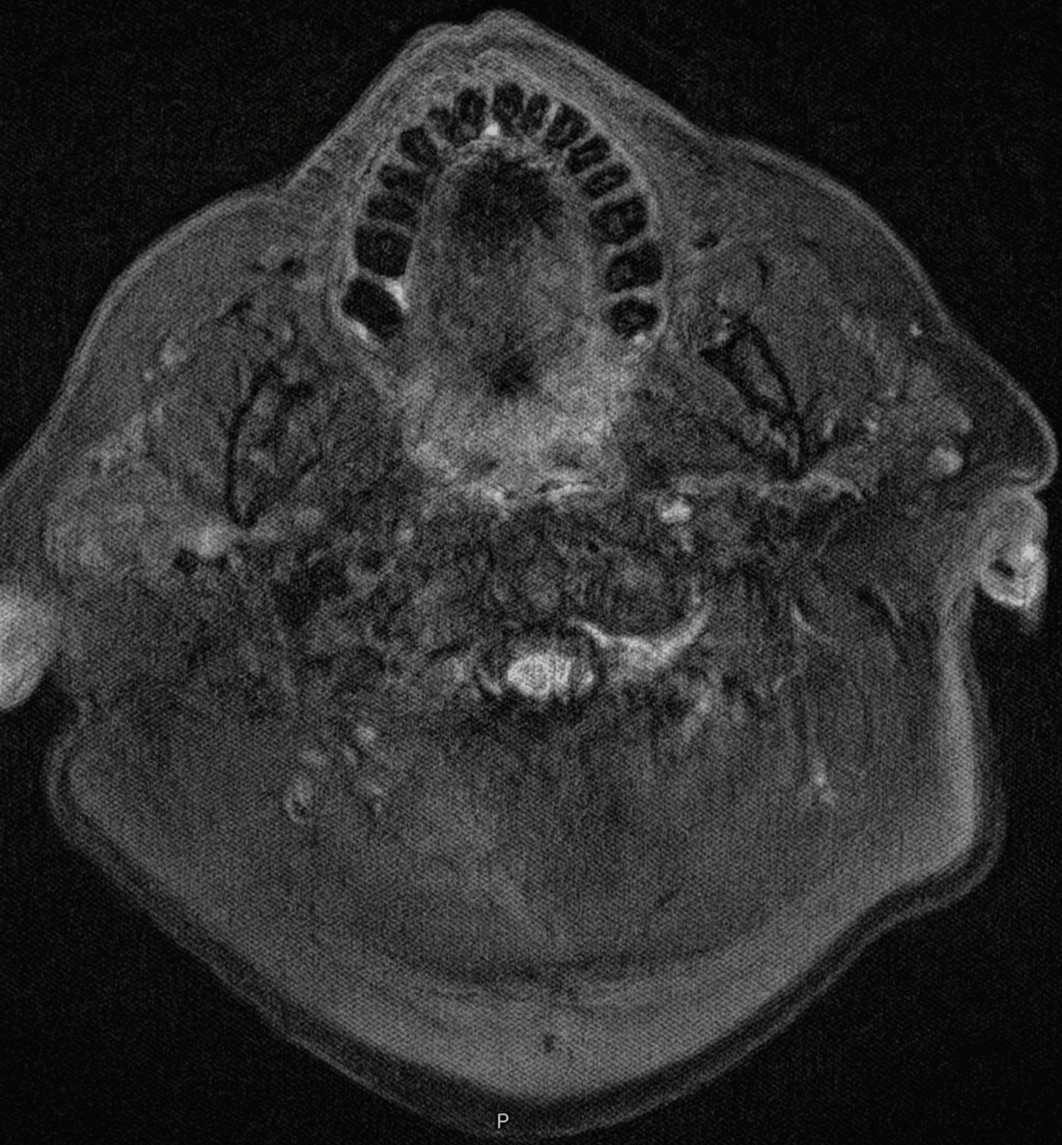
MRI of the brain showing no brain metastasis (T2-weighted FLAIR on an axial view) MRI: magnetic resonance imaging; FLAIR: fluid-attenuated inversion recovery

MRI of the brain revealed no metastatic disease (Figure 6). 

**Figure 7:**
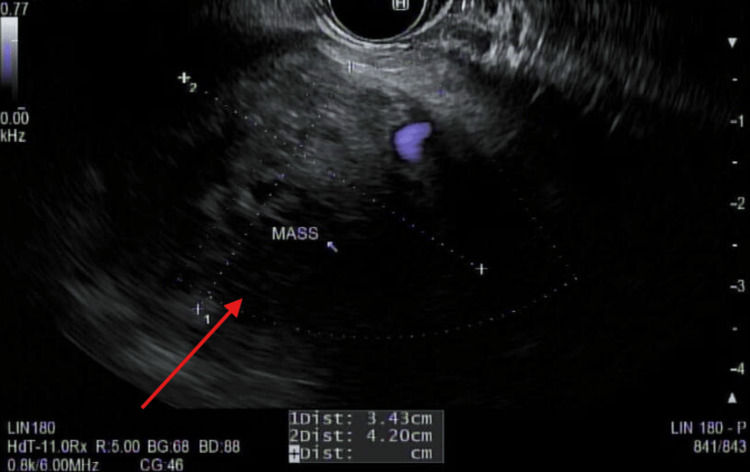
EUS revealing an oval, hypoechoic mass in the tail of the pancreas measuring 4.2×3.43 cm in maximal cross-sectional diameter Arrow highlights the well-defined margins of the hypoechoic pancreatic mass. EUS: endoscopic ultrasound

Endoscopic ultrasound (EUS)-guided biopsy of the pancreatic mass confirmed pancreatic adenocarcinoma (Figure 7), and the patient was discharged for outpatient oncologic follow-up. 

## Discussion

A pathologic fracture in the absence of significant trauma should raise suspicion for underlying malignancy, particularly when accompanied by systemic features such as weight loss or fatigue [5]. The differential diagnosis includes primary bone malignancies, hematologic cancers, and metastases from solid tumors such as the lung, breast, prostate, thyroid, and kidney [5]. Osteoporotic fractures typically occur in characteristic locations (vertebral bodies, proximal femur, distal radius) and are associated with diffuse bone demineralization rather than focal lytic destruction, cortical breach, or soft tissue involvement [6,7]. The presence of a focal lytic lesion with cortical destruction, as seen in this case, strongly favors a malignant process and should prompt the evaluation for occult malignancy [7].

Although pancreatic adenocarcinoma rarely metastasizes to bone, clinicians should not exclude it, especially in patients with risk factors such as tobacco use and alcohol consumption [2-4]. Skeletal metastases in pancreatic cancer are considered uncommon clinically, and reported cases often present at an advanced stage [3,8]. Appendicular metastases, such as the femur, are particularly rare [3,8]. One case report described a patient presenting with bone pain due to bone metastasis as an initial presentation in pancreatic ductal adenocarcinoma, illustrating the atypical nature of such presentations [8].

Lesions may be osteolytic, osteoblastic, or mixed, and their discovery often indicates advanced systemic disease with poor prognosis [5]. The presence of bone metastasis in pancreatic adenocarcinoma has been correlated with significantly reduced survival, particularly when associated with additional visceral metastases [9,10].

EUS-guided biopsy remains the gold standard for tissue diagnosis, allowing cytologic confirmation and molecular profiling [11-13]. In this case, EUS-guided fine-needle aspiration was critical for establishing the diagnosis, given the pancreatic tail location and its high diagnostic yield while simultaneously assessing local invasion and regional lymphadenopathy [12,13].

Our case adds to the limited number of reported instances where pancreatic cancer presented as a pathologic femoral fracture. Recognizing this rare presentation can help clinicians avoid diagnostic delay and direct appropriate investigations early. Management of such patients is primarily palliative and requires a multidisciplinary approach, including orthopedic stabilization for fracture management, systemic chemotherapy according to current guidelines, and consideration of palliative radiotherapy for symptomatic bone lesions [6,14]. Palliative radiotherapy, orthopedic stabilization, and systemic chemotherapy remain the mainstays of management, with an emphasis on symptom control and quality of life [6,14].

## Conclusions

This case underscores the critical importance of considering hidden malignancy when evaluating atraumatic fractures. Even in the absence of classical gastrointestinal symptoms, metastatic pancreatic cancer should be part of the differential diagnosis when imaging reveals destructive bone lesions. Early identification of atypical presentations allows the timely diagnosis and multidisciplinary management of patients with advanced pancreatic cancer.
